# A Rare Cause of Recurrent Febrile Encephalopathy in a Child: The Expanding Spectrum of ATP1A3 Mutations

**DOI:** 10.7759/cureus.20438

**Published:** 2021-12-15

**Authors:** Saja Tahir, Nidheesh Chencheri, Abdalla A Abdalla, Mohamed O E Babiker

**Affiliations:** 1 Pediatrics, Al Jalila Children's Speciality Hospital, Dubai, ARE; 2 Pediatric Neurology, Al Jalila Children's Speciality Hospital, Dubai, ARE; 3 Pediatric Neurology, Akron Children's Hospital, Akron, USA; 4 Pediatrics, Northeast Ohio Medical University, Rootstown, USA

**Keywords:** atp1a3, episodic encephalopathy, genetics, fever, hypotonia

## Abstract

ATP1A3 mutations have been recognized in infants and children presenting with a diverse group of neurological phenotypes, including rapid-onset dystonia parkinsonism (RDP), alternating hemiplegia of childhood (AHC), and cerebellar ataxia, areflexia, pes cavus, optic atrophy, and sensorineural hearing loss (CAPOS) syndrome. A new phenotype of fever-induced paroxysmal muscle weakness and encephalopathy (FIPWE) in patients with ATP1A3 mutations at c.2267G>A p residue 756H has been described most recently in few cases. Here, we report an additional case with an ATP1A3 mutation at c.2267G>A p residue 756H presenting with fever-induced paroxysmal muscle weakness and encephalopathy. To the best of our knowledge, this is the first reported case from the Middle East. This 18-month-old boy presented with recurrent, reversible fever-induced episodes of seizures, central hypotonia, areflexia, and developmental regression. The mainstay management for patients with ATP1A3 related diseases is symptomatic treatment as there is no specific proposed treatment. Aggressive management of febrile illness may be helpful in alleviating the symptoms.

## Introduction

Following the discovery of ATP1A3 mutations, multiple distinct phenotypes have been described. Most recently, a new phenotype in patients with ATP1A3 mutations at c.2267G>A p 756 has been reported in the literature [1]. The patients presented with fever triggered episodes of marked hypotonia, seizures, and some level of developmental regression. In between the attacks, patients regained skills or exhibit slow recovery [1]. The aim of this case report is to describe the clinical presentation and the management strategy of an additional case of ATP1A3 mutation at c.2267G>A p 756H.

## Case presentation

This 18-month-old boy, youngest of three siblings, was born at full term with a normal birth history. His development progressed normally until the age of four months when he presented with two episodes of generalized seizures during a febrile illness. He appeared lethargic and hypotonic. Possibility of intracranial infection was considered and infectious workup including cerebrospinal fluid analysis was normal. Magnetic resonance imaging (MRI) brain and electroencephalogram (EEG) were normal. He had a gradual recovery of symptoms over the following days.

At the age of nine months, during a febrile illness due to a viral upper respiratory tract infection (URTI), he presented with marked floppiness along with an episode of generalized tonic-clonic seizure. He was noted to have regression of motor milestones during this febrile episode. This prompted us to repeat the workup for possible infectious and metabolic causes which were unremarkable. He made a gradual recovery as the febrile illness resolved.

A month later, at the age of 10 months, he developed a similar episode following post-vaccination febrile illness. Within the same month, he had an additional episode of fever due to a URTI. This was again associated with generalized hypotonia, hyporeflexia, and choreiform movements along with regression of developmental milestones. He was not able to sit, not able to bear weight, and lost babbling. However, he made a slow recovery over the following three to four weeks.

In view of the recurrent fever-associated neurological symptoms with unclear etiology, whole exome sequencing was obtained. The sequencing identified a de novo mutation at ATP1A3 c.2267G>A pathogenic variant (p.Arg756His). During a subsequent febrile illness at the age of 14 months, aggressive management of his fever using regular administration of antipyretics and adequate hydration were done in addition to starting oxcarbazepine. By the end of the febrile illness, no developmental regression was noted. Currently, the patient is maintaining steady developmental progress, but he is still delayed. Oxcarbazepine was subsequently stopped as it was not felt to be of help.

## Discussion

Patients with ATP1A3-related disorders demonstrate variable phenotypic spectrum presenting with impairments in cognition, language, mood, behavior, gross motor, and fine motor functions [2]. In recent times, with the increased availability of genetic testing, the phenotypic spectrum of the disease has expanded. The spectrum evolved from the well-characterized clinical phenotypes of alternating hemiplegia of childhood (AHC), rapid-onset dystonia parkinsonism (RDP), and cerebellar ataxia, areflexia, pes cavus, optic atrophy, and sensorineural hearing loss (CAPOS) syndrome (cerebellar ataxia, areflexia, pes cavus, optic atrophy, and sensorineural hearing loss) to a newly described phenotype, fever-induced paroxysmal muscle weakness and encephalopathy (FIPWE) in patients with a novel mutation of ATP1A3 at c.2267G>A p residue 756H [1].

Patients with this mutation are reported to manifest a new clinical presentation in comparison to patients with other ATP1A3 mutations. These patients present with fever-induced encephalopathy as a key differentiating feature [1]. We report an additional case of FIPWE with ATP1A3 mutations at c.2267G>A p residue 756H. To the best of our knowledge, this is the first reported case from the Middle East. Our patient presented at early infancy with a clinical syndrome triggered by febrile illnesses. In each episode, marked encephalopathy, central hypotonia, developmental regression, and seizures were noted. This was followed by slow recovery after the resolution of the febrile illness. This clinical picture is similar to four previously described patients with c.2267G>A p 756H mutation and two other patients with c.2267G>A p 756L mutation [1,3,4].

FIPWE shares some of the clinical characteristics of other phenotypes of ATP1A3 mutations. Like CAPOS syndrome, FIPWE is triggered by hyperthermia. In addition, both groups of patients present with generalized and symmetric symptoms which can include gait ataxia and weakness. The symptoms of both conditions are reversible and get better as the fever and illness improve. However, in CAPOS syndrome, movement problems, vision changes, and sensorineural hearing loss may progress [5,6].

When comparing FIPWE to the previously described ATP1A3 mutation phenotypes, hemiplegia of childhood (AHC) and rapid-onset dystonia parkinsonism (RDP) have a completely different distinctive clinical presentation. AHC and RDP have been proposed to form a continuum, as they have significant phenotypic overlap with intermediate cases, and the same mutations can cause AHC and RDP [1]. They are often triggered by events such as running, alcohol binges, minor head injuries, overheating, and emotional stress [7]. AHC clinical features manifest with episodes of hemiplegia or quadriplegia improving by sleep. They also present with paroxysmal abnormalities such as dystonia, tonic spells, epileptic seizures, autonomic changes, and/or abnormal eye movements [7]. RDP presents with dystonia and parkinsonism features, which are permanent.

The mainstay management for patients with ATP1A3-related diseases is symptomatic treatment as there is no specific proposed treatment. However, flunarizine is used in cases with AHC [4]. Acetazolamide has been suggested in management of CAPOS syndrome [8]. The ATP1A3 gene encodes the alpha-3 subunit of the Na+/K+ ATPase pump which maintains the electrochemical gradients of sodium and potassium ions across the plasma membrane. It is possible that oxcarbazepine plays a positive role in this mechanism but our patient didn't seem to benefit from it. Since FIPWE is triggered by febrile illness, aggressive treatment of febrile illness might be a useful management strategy. Our patient seems to benefit from aggressive control of fever.

## Conclusions

Febrile encephalopathy is a common scenario in clinical practice with a wide differential diagnosis, including infectious and metabolic causes. ATP1A3 mutations-related disorders like FIPWE have been increasingly implicated in fever-related encephalopathies. Recurrent encephalopathy with unclear etiology warrants targeted sequence of ATP1A3 gene. High index of suspicion and awareness of such a unique clinical condition is required to make this rare diagnosis.
